# Sexually transmitted infections among migrants' wives remaining in rural homes – a pilot study of the remaining women in rural Wuhan, China

**DOI:** 10.1080/21642850.2013.872991

**Published:** 2014-01-21

**Authors:** Shanbo Wei, Xinguang Chen, Gang Li, Wang Zhou, Weidong Shi, Xia Wang

**Affiliations:** ^a^ Wuhan Family Planning and Health Commission, Wuhan, China; ^b^ Wuhan Centers for Disease Prevention and Control, Wuhan, China; ^c^ Wuhan Institute of Sexually Transmitted Diseases, Wuhan, China; ^d^ School of Medicine, Wayne State University, Detroit, MI, USA

**Keywords:** rural women, migrant workers, HIV risk behaviour, RTI, China

## Abstract

The growing HIV/AIDS epidemic in China appears to be related to the vast rural-to-urban migration, with rural migrants serving as a “living bridge” for the spread of HIV. The purpose of this study is to examine whether migrants' wives remaining in rural homes play a role in spreading the virus. Participants were recruited from 12 rural villages. Social and demographic factors, sexual behaviour, and HIV/AIDS knowledge were assessed using survey questionnaire. Reproductive tract infection (RTI; syphilis, chlamydia, gonorrhea, vaginalis trichomonas, and candidiasis) were assessed using blood and vaginal specimens. Among the total 63 participants, 28 (44.4%) were wives remaining behind while their husbands migrated to a city (“remaining”) and 35 were women whose spouses remained in the rural setting (“comparison”). The reported median duration (inter-quarter range (IQR)) since the last episode of sex with husband was nine months (IQR: 7–15) for the remaining women and three months (IQR: 2–7) for the comparison women (*Z* = 3.95, *p* < 0.01). RTI was 32.1% for remaining women and 17.1% for the comparison women (Odds ratio = 2.28, 95%CI: 0.70–7.48, *p* = 0.165). The high rate of RTI suggests that remaining women in rural areas may be at increased risk for acquiring HIV infection compared to women whose husbands remained in rural homes.

## Introduction

The growing HIV/AIDS epidemic in China appears to be related to the vast rural-to-urban migration, with rural migrants serving as a “living bridge” for the spread of HIV (Chen, Stanton, Li, Fang, & Lin, 2008; Chen et al., 2009, 2011; Lau et al., 2013; Li et al., 2004, 2007). Among HIV-positive individuals in China, a disproportionately greater number are rural residents and rural-to-urban migrants (Hesketh et al., 2006; Xiao et al., 2010). Many married men at the reproductive age have migrated to work in the city and left their spouses at home (Zhang, Chow, Jahn, Kraemer, & Wilson, 2013). Studies have shown that HIV risk behaviours, such as substance use and sexual risk behaviours are common among rural-to-urban migrants, including married males who migrate alone (Chen et al., 2009; Lin et al., 2005; Wang et al., 2007; Zhang et al., 2013). If a migrant man has contracted HIV infection during his urban stay, he may transmit the virus to his wife through sex when he returns to his rural home.

Married women who remain behind in rural homes may also be more likely than women living with their spouses at rural homes to engage in sexual risk behaviours, passing the AIDS virus to others who are not infected and/or acquiring the virus from those who are infected in rural areas (Chen et al., 2008, 2009; Li et al., 2007; Zhang et al., 2013). Although much has been documented regarding HIV risk behaviours among rural-to-urban migrants in urban China (Chen et al., 2008, 2009; Li et al., 2004, 2007; Zhang et al., 2013), few reported studies ever examined HIV risk among rural wives remaining behind when their husbands migrate. Compared to their urban counterparts, rural Chinese are relatively poor, have received limited education, and have few opportunities to be exposed to knowledge and prevention regarding HIV/AIDS (Lau et al., 2013; Ma, Wang & Zheng, 2003; Qian et al., 2006; Wang et al., 2007). Data are highly needed regarding risk of HIV infection among the vulnerable and underserved rural residents, particularly wives of rural-to-urban migrants who remain in the rural settings.

We speculated that married women remaining behind in their rural homes may play a critical role in bridging the spread of HIV/AIDS through population migration. However, due to the challenge to access rural women, data are relative scant regarding HIV and other related reproductive tract infections (RTI) among rural women. Data from a few early studies suggest that some RTIs, such as syphilis, chlamydia, vaginalis trichomonas, and candidiasis, may be more common among women in rural areas than in urban areas (Ma et al., 2003; Xia, Liao, He, Choi, & Mandel, 2004). In this study, we compared the sexual behaviours and rates of RTI among a sample of women who remained at rural homes while their spouse went to work in cities with women whose spouses stay at home. Data used for this analysis were derived from a pilot study.

## Methods and materials

Participants of this study included married women who remained at home in rural villages, while their husbands migrated to work in the city (“remaining wives”). Women in the same village whose husbands did not migrate were included as a comparison group (“comparison wives”). Participants (one per household) were sampled from 63 households in 12 natural villages from Shanpo Township, Jiangxia County of Wuhan. The Shanpo Township is well known for its high proportion of farmers migrating to work in cities. Villages were sampled randomly using the simple random digits method.

After a village was sampled, the investigators from Wuhan Centers for Disease Prevention and Control (CDC) travelled to the site via a van. The van is equipped as the Mobile Clinic, including space for survey administration and a bed for biological sampling. The trained data collectors (one preventive physician companied by one nurse) went door to door to recruit participants using the Institutional Review Board (IRB) approved informed consent protocol. Assistance was obtained from the local Village Chinese Women Association (a grass root non-government organization of women in China) to locate households with potential candidates.

All married women below 50 years of age in the villages were eligible to participate if their husbands were working in the city at the time of the study and they did not migrate to urban areas in the past three years. The same age range and migration criteria were used to recruit comparison women in the same village where a remaining woman was sampled. Among all the eligible women approached, approximately a half agreed to participate.

After providing informed consent, the participants were brought to the Mobile Clinic to complete data collection. Data regarding social and demographic factors (age, educational attainment, annual family income in Chinese Yuan, and number of children), duration since the last sex with spouse (months), and sexual risk behaviours (having sex with partners other than spouse, y/n; condom use during sex, y/n). Data regarding HIV knowledge were collected through in-person interview using a structured questionnaire. Levels of HIV knowledge were assessed using the HIV Knowledge Scale (12 items, Cronbach alpha = 0.85) that has previously been validated and used in China (Yan, Liu, & Wu, 2003). Typical statements in the scale include: “Is AIDS an infectious disease?”; “Can a person be infected by eating together with an AIDS patient?” and “Can a person be infected by shaking hands with an AIDS patient or a person who is tested HIV positive?” Answer options were: “yes”, “no”, and “don't know”. One point was acquired for each correct answer; a score was created by summing the number of correct answers such that higher scores indicating greater knowledge regarding HIV/AIDS.

Blood samples (5 ml per person) were collected for testing syphilis (screen test by TRUST, Shanghai Rongsheng Biotech Co Ltd, and confirmatory by TPPA, Fujrebio Inc) and chlamydia (PCR, Roche Diagnostics Ltd). Vaginal specimens were collected for testing gonorrhea (T-M culture medium, Oxoid Ltd), and Vaginalis trichomonas and candidiasis (smear test using a microscope). The smear tests for vaginalis trichomonas and candidiasis were conducted in the Mobile Clinic on site after the sample was collected. The biological samples for testing Syphilis, Gonorrhea, and Chlamydia were transported to Wuhan CDC on a daily basis and the tests were conducted at a designated laboratory in Wuhan CDC.

Survey administration and biological sampling were completed by a trained and certified female preventive physician with experience working with rural women. Participants received a gift equivalent to $3 in value after completing data collection. Approval of the study protocol was obtained from the Wuhan IRB.

All data were manually entered into computer. Descriptive statistics such as mean, standard deviation (SD), median, and inter-quarter range (IQR), rate and ratio were used to summarize the characteristics of the study sample. Comparative analyses, including student *t*-test (for continuous variables), *χ* ^2^ test (for categorical variables) were used to assess if the remaining women were at increased risk of HIV infection. Fisher's exact test was used for categorical variables with the expected frequency < 5. Wilcoxon–Mann–Whitney test was used for continuous variables that were non-normal (e.g. time since the last sex with spouse). Statistical analysis was conducted using the software SAS version 9.2 (SAS Institute, Inc., Cary, NC).

## Results

Among the total 63 participants, 28 were remaining wives and 35 were comparison wives (Table 1). The remaining wives were younger than the comparison wives (37.0 years *vs.* 41.1 years, *p* < 0.05). Overall, more than half of the rural women had a primary level education with a median family income of 6000 RMB (approximately $755) per year. Approximately, 80% of these rural women reported having two or more children. The reported median duration (months) since last sex with spouse was significantly longer for the remaining wives than for the comparison women (9 *vs.* 3, *Z* = 3.95, *p* < 0.01 from the Wilcoxon–Mann–Whitney test).

**Table 1.** Comparison of remaining women whose husband is working in the city with women whose husbands staying in rural home, Wuhan, China.

Variable	Remaining women	Comparison women	Total	Statistic (*p* value)
*N*	28 (44.4%)	35 (55.6%)	63 (100%)	
*Age in years*				
Range	23–47	27–53	23–53	
Mean (SD)	37.0 (4.8)	40.1 (5.9)	38.7 (5.6)	*t* = 2.25 (0.028)
*Education*				
≤Primary school	14 (50.0%)	21 (60.0%)	35 (55.6%)	
≥Middle school	14 (50.0%)	14 (40.0%)	28 (44.4%)	*χ*^2^ = 0.63 (0.427)
*Family income*				
<3000	5 (17.9%)	11 (31.4%)	16 (25.4%)	
3000–	12 (42.9%)	14 (40.0%)	26 (41.3%)	
6000–	11 (39.3%)	10 (28.6%)	21 (33.3%)	
Median	5000	5000	5000	*χ*^2^ = 1.69 (0.429)
*No. of children*				
1	9 (32.1%)	4 (11.4%)	13 (20.6%)	
2	14 (50.0%)	16 (45.7%)	30 (47.6%)	
3–	5 (17.9%)	15 (42.9%)	20 (31.8%)	*χ*^2^ = 6.36 (0.042)
*Time since last sex with spouse*				
Median	9 months	3 months	5.5 months	*Z* = 3.60^a^
Q1–Q3	7–15	2–7	2–10	(0.0003)^a^
*Use condom during the last sex*				
Yes	3 (10.7%)	0	3 (4.8%)	Fisher test
No	25 (89.3%)	35	60 (95.2%)	(0.087)^b^
*Have sex with persons rather than spouse*				
Yes	1 (3.6%)	0	1 (1.6%)	Fisher test
No	27 (96.4%)	35	62 (98.4%)	(0.452)^b^
*HIV knowledge*				
Mean (SD)	5.6 (3.3)	4.8 (3.7)	5.1 (3.5)	*t* = 0.91 (0.368)
RTI^c^				
Positive	9 (32.1%)	6 (17.1%)	15 (23.8%)	
Negative	19 (67.9%)	29 (82.9%)	48 (76.2%)	*χ*^2^ = 1.93 (0.165)

^a^Wilconxon–Mann–Whitley nonparametric test.

^b^Fisher's exact test.

^c^RTI, reproductive tract infection, including a positive result from any of the follow five infections (syphilis, chlamydia, gonorrhea, vaginalis trichomonas, and candidiasis) that were commonly reported in China. OR = 2.29 (95% CI: 0.70–7.48, *p* = 0.165, two-sided) for positive RTI among women whose husbands went to work in the city as compared to women whose spouses stayed at home.

Among the 28 remaining wives, 3 reported using condom during the most recent episode of sex while none of the 35 comparison wives reported using a condom. One remaining wife reported having sex with persons other than her spouse and none of the 35 comparison wives reported such behaviour. The mean HIV knowledge score was 6.14 (SD = 3.09) for the remaining wives and 5.14 (SD = 3.39) for the comparison wives.

Among the total 63 participants, laboratory tests found 4 positive for Chlamydia, 2 positive for vaginalis trichomonas, 1 positive for syphilis, and 9 positive for candidiasis. No sample tested positive for gonorrhea. The prevalence rate of any of these RTIs was 32.1% (9/28) and 17.1% (6/35) for the remaining wives and the comparison wives, respectively. Among the 28 remaining wives, one tested positive for both Chlamydia and Vaginalis trichomonas and no such double-infection was detected among the 35 comparison women. The odds ratio (OR) of laboratory-detected RTI was 2.29 (95% CI: 0.70–7.48, *p* = 0.16, two-sided, 0.08, one-sided) for the remaining women relative to the comparison women.

## Discussion

In this article, we report results from a pilot survey study to assess RTI, HIV knowledge, and HIV risk and protective behaviours among a sample of married women in a rural area in Wuhan, China. Compared to other married women in the same rural villages staying with their husbands at home, wives whose husbands migrated to cities tend to be younger with fewer children, reported longer duration of not having sex with husbands. The laboratory detected prevalence of RTI was about twice as high for the remaining women compared to the comparison women. Data from diverse sources indicate that the prevalence rate of sexually transmitted infections among high risk female sex workers (FSWs) and men-having-sex-with men (MSMs) varied between 17% and 35% (Chen, Peeling, Yin, & Mabey, 2011; Zhang et al., 2013). If the finding of our study can be confirmed with larger samples, it would mean that the risk for HIV infection among the remaining women in rural areas may be as high as that for FSWs and MSMs in China. Findings of this study provide useful data supporting future studies with adequate statistical power to investigate HIV risks among remaining women in rural China for more effective HIV prevention.

Fortunately, despite the potential higher risk of RTI infection, the remaining women appear to be more knowledgeable about HIV/AIDS and are more likely to use a condom during sex. It could be that these women have more time and resources to access related knowledge and facilities through their spouses. If this is the case, it will provide opportunities to delivery HIV prevention intervention programs to these women. Additional studies are suggested to investigate these protective factors in order to protect vulnerable remaining wives in rural settings (Luo & He, 2008). Findings of our study also suggest that the increases in the HIV knowledge and HIV protective behaviour among these rural women are not very high in an absolute term. This implies barriers for these rural women to obtain HIV knowledge and to receive guidance to practice HIV protective behaviours for purposes of prevention.

This study is important because few studies ever used laboratory data to document RTIs among rural women in China in general, not to mention the remaining women. Up to date, much effort has been devoted to FSWs and MSMs, and very little attention has been paid to millions of remaining women in the countryside, while their spouses are away in urban areas. Findings of this pilot study support the two mechanisms as we proposed for the role of the remaining rural women in spreading the HIV virus. (1) Remaining women may have acquired the RTIs from their husbands who bring the infection from urban areas back home. If this is the case, an HIV-infected husband may certainly be able to pass the virus to his wife reaming in the rural home if they have sex without using a condom. (2) Remaining women may also acquire the infection by engaging in sexual risk behaviours in rural areas. Our data showed that the last time to have sex with spouse was nine and three months for the remaining women and for the comparison women, respectively. One reason for the higher rates of RTI among the remaining wives could be that these women engaged in risky sexual behaviours while their husbands are not at home. Further studies are needed to examine the role of these two related mechanisms in bridging the rural-urban HIV spread in China.

Every year, more than 200 million people in China move from rural areas to cities to earn income and most of the married men move alone, leaving their spouse in rural homes to maintain their families and to take care of their old parents and young children. Any data on HIV risk among these women is of great significance for HIV/AIDS prevention and control. Despite the small sample size that has limited this study to support the high OR of HIV risk for the remaining rural women, data reported in this study provided useful information supporting the advancement of current HIV research to include remaining wives in the migration-related HIV spread in China – an urgent but relatively less studied issue. Further research is indicated to verify the findings observed in this study to support more effective strategy for HIV prevention in China.

## References

[CIT0001] Chen X., Stanton B., Kaljee L., Fang X., Xiong Q., Lin D., Li X. (2011). Social stigma, social capital reconstruction and rural migrants in urban China: A population health perspective. *Human Organization*.

[CIT0002] Chen X., Stanton B., Li X., Fang X., Lin D. (2008). Substance use among rural-to-urban migrants in China: A moderation effect model analysis. *Substance Use & Misuse*.

[CIT0003] Chen X., Stanton B., Li X., Fang X., Lin D., Xiong Q. (2009). A comparison of health-risk behaviors of rural migrants with rural residents and urban residents in China. *American Journal of Health Behavior*.

[CIT0004] Chen X. S., Peeling R. W., Yin Y. P., Mabey D. C. (2011). The epidemic of sexually transmitted infections in China: Implications for control and future perspectives. *BMC Medicine*.

[CIT0005] Hesketh T., Li L., Ye X., Wang H., Jiang M., Tomkins A. (2006). HIV and syphilis in migrant workers in eastern China. *Sexually Transmitted Infections*.

[CIT0006] Lau J. T., Yu X., Mak W. W., Cheng Y., Lv Y., Zhang J., Wang Z. (2013). Prevalence of inconsistent condom use and associated factors among HIV discordant couples in a rural county in China. *AIDS and Behavior*.

[CIT0007] Li X., Fang X., Lin D., Mao R., Wang J., Cottrell L., Stanton B. (2004). HIV/STD risk behaviors and perceptions among rural-to-urban migrants in China. *AIDS Education and Prevention*.

[CIT0008] Li X., Zhang L., Stanton B., Fang X., Xiong Q., Lin D. (2007). HIV/AIDS-related sexual risk behaviors among rural residents in China: Potential role of rural-to-urban migration. *AIDS Education and Prevention*.

[CIT0009] Lin D., Li X., Yang H., Fang X., Stanton B., Chen X., Liu H. (2005). Alcohol intoxication and sexual risk behaviors among rural-to-urban migrants in China. *Drug and Alcohol Dependence*.

[CIT0010] Luo Y., He G. P. (2008). Pregnant women's awareness and knowledge of mother-to-child transmission of HIV in south central China. *Acta Obstetricia et gynetologica Scandinvaica*.

[CIT0011] Ma C. L., Wang G. Q., Zheng Y. J. (2003). STD prevalence in the general female population. *Chinese Journal of AIDS & STD*.

[CIT0012] Qian H. H., Vermund S. H., Kaslow R. A., Coffey C. S., Chamot E., Yang Z., Wang N. (2006). Co-infection with HIV and hepatitis C virus in former plasma/blood donors: Challenge for patient care in rural China. *AIDS*.

[CIT0013] Wang B., Li X., Stanton B., Fang X., Liang G., Liu H., Yang H. (2007). Gender differences in HIV-related perceptions, sexual risk behaviors, and history of sexually transmitted diseases among Chinese migrants visiting public sexually transmitted disease clinics. *AIDS Patient Care STDS*.

[CIT0014] Xia D. Y., Liao S. S., He Q. Y., Choi K. H., Mandel J. S. (2004). Self-reported symptoms of reproductive tract infections among rural women in Hainan, China: Prevalence rates and risk factors. *Sexually Transmitted Diseases*.

[CIT0015] Xiao Y., Sun J., Li C., Lu F., Allen K. L., Vermund S. H., Jia Y. (2010). Prevalence and correlates of HIV and Syphilis infections among men who have sex with men in seven provinces in China with historically low HIV prevalence. *Journal of Acquired Immune Deficiency Syndromes*.

[CIT0016] Yan J. Y., Liu Z. F., Wu X. H. (2003). KAP about AIDS among rural women in China. *Chinese Preventive Medicine*.

[CIT0017] Zhang L., Chow E. P., Jahn H. J., Kraemer A., Wilson D. P. (2013). High HIV prevalence and risk of infection among rural-to-urban migrants in various migration stages in China: A systematic review and meta-analysis. *Sexually Transmitted Diseases*.

[CIT0018] Zhang X., Yu J., Li M., Sun X., Han Q., Li M., Gao L. (2013). Prevalence and related risk behavior of HIV, syphilis, and anal HPV infection among men who have sex with men from Beijing, China. *AIDS and Behavior*.

